# Prevention and management of external fixator pin track sepsis

**DOI:** 10.1007/s11751-012-0139-2

**Published:** 2012-06-23

**Authors:** Nando Ferreira, Leonard Charles Marais

**Affiliations:** Tumor, Sepsis and Reconstruction Unit, Department of Orthopaedic Surgery, Greys Hospital, University of KwaZulu Natal, Private bag X9001, Pietermaritzburg, 3201 South Africa

**Keywords:** Pin site, Infection, Complications, External fixation

## Abstract

Pin track-associated complications are almost universal findings with the use of external fixation. These complications are catastrophic if it leads to the failure of the bone–pin interface and could lead to pin loosening, fracture non-union and chronic osteomyelitis. Strategies proposed for the prevention and management of pin track complications are diverse and constantly changing. Prevention of external fixation pin track infection is a complex and ongoing task that requires attention to detail, meticulous surgical technique and constant vigilance.

## Introduction

External fixation is an essential component of the modern orthopaedic surgeon’s armamentarium and is widely used in traumatology and reconstructive surgery. This treatment modality is unfortunately associated with the almost universal complication of pin track infection [1, 2].

This article aims to highlight the factors associated with an increased risk of pin track complications, reviews the literature and proposes a protocol for effective external fixator pin track care.

## Background

Pin track infection is almost inevitable during the long-term use of external fixators with the quoted incidence ranging from 11.3 to 100 % [3–11]. Bibbo [2] stated that ‘Pin-site irritation/infection have almost become an accepted certainty in the realm of external fixation, with physicians relying heavily on the majority of those complications resolving without consequences by using appropriate pin care and antibiotic therapy’.

## Fixator pin–bone interface stability

Pin track infection decreases the stability of the pin–bone interface. Conversely, instability of the fixator pin–bone construct can lead to half-pin loosening and infection [3]. It is a common misconception that pin loosening only results from pin track infection when in actual fact pin loosening is often the initiating event resulting in pin track sepsis.

In the light of this, the external fixator construct is crucial in the prevention of pin track infection. The overall stability of the external fixator construct is the result of a complex interplay of variables. The forces transmitted through the fixator and limb is a function of the geometrical and mechanical properties of the fixator as well as the properties of the surrounding tissues and the fracture pattern [12]. There is, also, what appears to be a race between the gradual increasing loading capacity of healing bone and potential failure of the bone–pin interface [13]. For this reason, it is important to keep the fracture configuration in mind when deciding on which external fixator to use.

An unstable fixator creates an unsuitable environment for optimal bone healing and leads to increased movement at the fixator pin–bone interface, producing pin site irritation and infection [3, 14]. Parameswaran et al. [3] found that the type of fixator had an effect on the incidence of pin site infection, with monolateral and hybrid fixators showing a much higher incidence of pin site infection than ring fixators.

In addition to a stable fixator construct, stable pin fixation is needed to prevent the vicious cycle of pin loosening, pin site infection and further loosening [15]. Moroni et al. [16] found that deterioration of bone–pin interface strength was an inevitable phenomenon with standard, uncoated pins. This was due to fibrous tissue formation at the bone–pin interface of uncoated pins, which led to loosening [17, 18]; this was recorded as a lower extraction torque force needed during pin extraction than was the insertion torque [9]. In contrast, hydroxyapatite-coated pins show improved fixation strength, with extraction torque forces being higher than the initial insertion torque forces and 90 times higher than standard uncoated pins [9]. This improved fixation translated into significantly lower rates of osteolysis; an 18 times lower incidence of pin loosening [9] and a decrease in pin site infection when compared to uncoated pins [11, 17–25]. At our institution, we have abandoned the use of uncoated pins in long-term external fixators.

## Pin insertion

It should be emphasized that any strategy for reducing pin site complications begins in the operating theatre [10]. Wire and pin insertion should be as low energy and atraumatic as possible, with minimum damage to the skin, soft tissue and bone.

Skin incisions should be placed with care, in order to avoid tension on the skin. At the same time, the incisions should only be as large as the diameter of the pin. Large open wounds surrounding pins should be avoided, and we recommend suturing unnecessarily large wounds around pins. The aim is to facilitate rapid healing of the skin around the pin or wire, in order to create a bone–pin interface that is sealed from the external environment.

In order to prevent damage to the soft tissue envelope, wires must be pushed onto bone and not drilled through the soft tissues. The location of the pin or wire placement must also be considered. Soft tissue movement around pins and wires leads to increased risk for infection [2, 26] and any pins located in areas with considerable soft tissue, tendons and tendon sheaths are at greater risk for infection [27]. To prevent transfixing muscles in a shortened position, any muscle compartment that is traversed should be placed under stretch during the placement of the pins and wires [2].

Heat generation must be guarded against during pin or wire insertion, as this could lead to thermal necrosis of the surrounding bone, ring sequestra and pin loosening. For this reason, the anterior tibial crest must be avoided, as drilling through the thick cortical bone can generate excessive heat [2]. In order to prevent heat generation during wire insertion, cortices are breeched via drilling and the wire is then advanced through the distal soft tissues with a mallet [5].

**Fig. 1:**
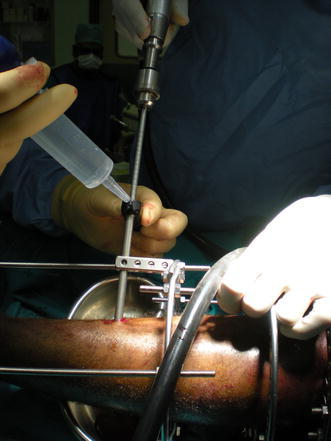
Cooling of drill while pre-drilling

**Fig. 2:**
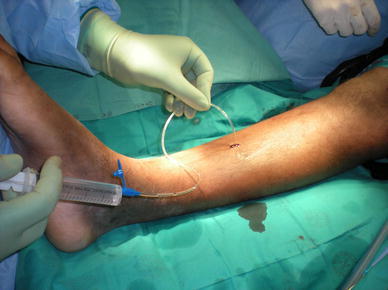
Irrigation of drill holes

**Fig. 3:**
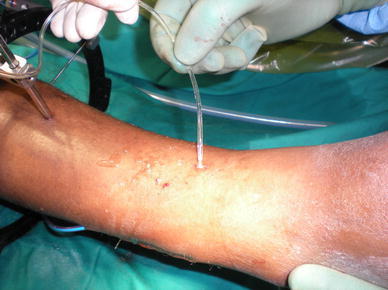
Bone swarf rinsed from drill tract

For half-pin placement, predrilling should always be performed even when using self-drilling pins [2, 5]. Drilling should be done in a pulsed (stop–start)/metronomic fashion together with continuous irrigation with cold saline to ensure proper pin cooling [2, 10] (Fig. 1). After drilling, the pilot hole must be irrigated to remove the bone swarf that might act as sequestra and prevent optimal bone–pin fixation [10] (Figs. 2, 3).

**Fig. 4:**
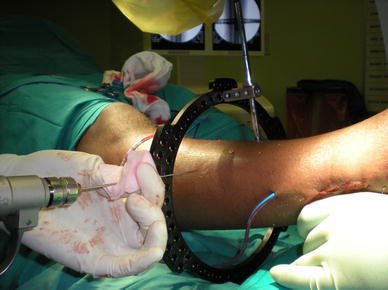
Non-touch insertion of wire

We adhere to the recommendations by Davies, and as far as possible use a non-touch technique when inserting half-pins [10]. To ensure a non-touch technique for inserting wires, we use chlorhexidine-soaked swabs to handle and manipulate wire placement (Fig. 4).

## Peri-operative management

Pin sites should be encouraged to heal around the wires and pins, like a pierced ear heals.^1^ After completion of the procedure, all pin sites must be free of skin tenting and soft tissue impingement [2, 5, 26]. Sterile dressings should be placed around pin sites and held continuously in place with a small amount of pressure, to prevent skin tenting and haematoma formation [28]. Various dressings have been used, ranging from dry dressings [28], open-cell foam dressing [2], betadine-soaked gauze [5], to alcoholic solution of chlorhexidine-soaked gauze [10]. Regardless of the choice of dressings, their main purpose is to keep the pin sites clean and dry, and absorb any blood and exudates [28] and therefore we discourage the usage of paraffin gauze around the pins.

**Fig. 5:**
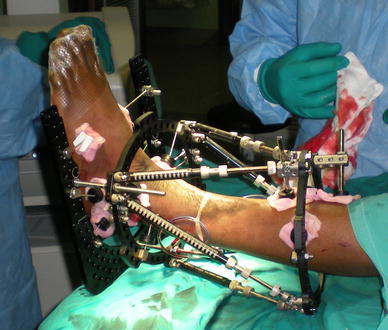
Pin sites dressed with chlorhexidine–alcohol solution swabs and slight pressure

**Fig. 6:**
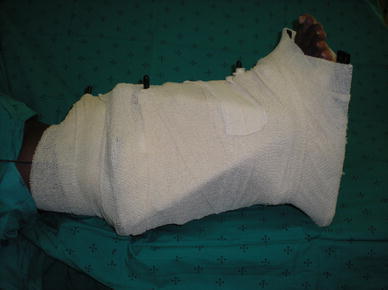
Post-operative dressing of fixator

In our unit, we follow the procedure described by Davies, who found lower infection rates when pin sites were dressed immediately after pin insertion with an alcoholic solution of chlorhexidine with pressure to reduce haematoma formation around pins (Fig. 5). These dressings are then changed at the end of the procedure if they are blood stained [10]. We also cover the whole limb and external fixator with a sterile dressing at the end of the procedure, and this dressing is left in place for the first post-operative week [31] (Fig. 6).

## Pin site care

There is no universally accepted protocol for the optimal care of pin sites [5]. In the absence of clear research evidence, consensus meetings have sought to provide guidance on pin site care. One such meeting was the Royal College of Nursing meeting held in the United Kingdom in 2010, which published their guidelines in 2011 [32]. In lieu of this, there are still a myriad of protocols available, ranging from a nihilistic approach with no active pin site care [29], to twice daily cleaning and dressings plus oral antibiotics for the entire duration of the external fixator [3].

The appropriate time to commence pin track care vary greatly in the literature with published times ranging from 24 h to 10 days [2, 3, 5, 10, 27–29, 31]. The frequency of pin track cleaning also differ, with authors suggesting once daily [6, 27], twice daily [3, 4], weekly [27, 33] or ‘when required’ [28].

Various cleaning solutions are advocated in the literature, including soap and water, sterile water, normal saline, peroxide, polyvinylpyrrolidone iodine, isopropyl alcohol and chlorhexidine [2–6, 10, 27, 28, 30]. When comparing chlorhexidine to normal saline, W-Dahl [30] found that chlorhexidine resulted in fewer positive bacteria cultures, lower frequency of *Staphylococcus aureus* and fewer days of antibiotic use.

We have however noted a small number of cases of chlorhexidine sensitivity resulting in skin irritation and weeping pin tracks. This finding is supported by Davies who reported a 17.6 % incidence of hypersensitivity reactions to prolonged skin contact with a strong antiseptic solution [10]. Fortunately, this usually resolves through the substitution of chlorhexidine with a mild soap and water solution for pin site care.

Dressing after pin track care is also controversial. Parameswaran et al. [3] used gauze packing with one to two drops per pin of a benzoalkonium chloride antiseptic solution. The Epic 2 guidelines used in an NHS hospital prescribe clear polyurethane (Allevyn™) dressings that are changed every 7 days [33]. Lee et al. [34] showed a decrease in pin site infection when comparing gauze impregnated with polyhexamethylene biguanide and plain gauze wet with saline. Davies advocates that pin sites are cleaned daily for the first 3 days, followed by alcoholic solution of chlorhexidine dressings. After day three, an occlusive dressing is applied and changed every 5–7 days [10]. Rose [5] reported that in the presence of exudates, pins should be dressed with gauze, but left uncovered in the absence of an exudate.

At our institution, a gauze swab with an alcoholic solution of chlorhexidine dressing is applied and left undisturbed for the first 7 days, followed by twice daily cleaning with a chlorhexidine solution. No pin site dressings are used once the pin sites have healed. Twice daily pin site cleaning is continued for the entire duration of the external fixation.

Another important preventative measure involves post-operative limb elevation. We advocate limb elevation whenever the patient is not actively mobilizing. This reduces oedema around the pins and creates the optimal environment for rapid healing of the pin tracks [2].

Showering is recommended, once the pin sites have healed, but thorough drying of the skin and the external fixator is mandatory thereafter. We do not advise swimming, but if a patient does insist, swimming in a chlorinated pool is permitted. No swimming in dams or in the ocean is allowed.

## Pin site infection

Pin site infections usually start as cellulitis around the pin or it may start as a localized form of osteitis, and most are secondary to *Staphylococcus aureus* infection, followed by *Pseudomonas aeruginosa* [9, 10]. Although there is no standardized system for classifying pin site infections [5], the Checketts-Otterburn classification is commonly used and provides valuable information regarding treatment [35] (Table 1). According to this system, pin site infections are classified into two groups, minor (Grades 1–3) and major (Grades 4–6), with the significant difference between the two groups being that the external fixation pin has to be abandoned in major infections [35].

**Table 1:** Checketts–Otterburn classification

Grade	Characteristics	Treatment
Minor infection		
1	Slight redness and little discharge	Improved pin site care
2	Redness of the skin, discharge, pain and tenderness in the soft tissue	Improved pin site care and oral antibiotics
3	Grade 2 but no improvement with oral antibiotics	Affected pin or pins resited and external fixation can be continued
Major infection		
4	Severe soft tissue infection involving several pins, sometimes with associated loosening of the pin	External fixation must be abandoned
5	Grade 4 but radiographic changes	External fixation must be abandoned
6	Infection after fixator removal. Pin track heals initially, but will subsequently break down and discharge in intervals. Radiographs show new bone formation and sometimes sequestra	Curettage of the pin tract

Although pin track infection is common, very few lead to major complications [2, 5, 7, 10]. Schalamon et al. [7] found that 94 % of infections were mild and responded to local or systemic antibiotic management. Piza also reported that 75 % of their pin site infections were minor infections when using the Checketts–Otterburn classification [9, 35]. Once pin site infection has been diagnosed, limb elevation is crucial as limiting the time that the limb is spent in a dependent position may help to hasten pin site quiescence [2]. Most authors advocate oral antibiotics directed against *Staphylococcus aureus* once pin site infection is diagnosed [2, 7, 29]. Bhattacharyya [36] found that nanocrystalline silver-releasing dressings were as effective as oral antibiotics to control pin site infection.

We advocate that pin track care is restarted as soon as pin site infection is identified. This includes twice daily cleaning of the pin–skin interface with a chlorhexidine solution and absorbent dressings if excessive exudate is encountered. A course of oral antibiotics aimed at *Staphylococcal* infection is prescribed for 7–10 days. Checketts grade 3 infections are admitted for intravenous antibiotics and in-hospital pin track care and limb elevation. If these infections do not respond adequately, the involved pins or wires are removed or exchanged.

## Pin removal

Major pin track infections, Checketts grade 4 and above, should be managed in theatre in order to allow adequate debridement of the pin tracks. Morgan-Jones [37] recommends arthroscopic debridement of major pin track infection to remove all necrotic debris. Bibbo [2] on the other hand, uses the Versajet Hydrosurgery system (Smith & Nephew, Memphis, TN) to debride infected pin sites after which the wound edges are freshened and closed with nylon or polypropolene sutures.

Bibbo also identified risk factors for developing non-healing wounds after pin removal, and these include: patients with diabetes mellitus, chronic venous insufficiency, peripheral vascular disease and poor soft tissue envelope due to trauma [2]. In these cases, it may even be necessary to raise small random-pattern fasciocutaneous flaps in order to treat non-healing pin sites [2].

In cases of osteomyelitic pin tracks with a sizeable cavity following debridement, these cavities can either be treated by leaving a 2-mm antibiotic bead in the track [3] or by using antibiotic-impregnated absorbable calcium-sulphate pellets to back-fill these tracks [2].

It is important to emphasize that pin or wire removal should not destabilize the frame construct as this will result in increased movement at the fixator pin–bone interface of the remaining pins and wires, initiating loosening and infection of the remaining pins [3, 14]. Therefore, septic pins and wires should, as a rule, rather be resited than simply removed.

## Conclusion

Pin site infection is a very common complication with external fixation. In an effort to prevent or at least minimize this complication, a pin site strategy should be adopted that covers all aspects associated with pin loosening and infection. This should include understanding of external fixator biomechanics, meticulous surgical technique during pin and wire insertion and a standardized post-operative pin site care protocol.

## Footnotes

1 Connecticut center for orthopedic surgery external fixator pin care protocol.
